# Emergency medical services in Armenia: national call trends and future directions

**DOI:** 10.1186/s12245-024-00644-y

**Published:** 2024-05-16

**Authors:** Ani Arzoumanian, Anya Agopian, Marine Hovhannisyan, Sharon Chekijian, Aline Baghdassarian

**Affiliations:** 1 Department of Psychological and Brain Sciences, Colgate University, Hamilton, USA; 2 Turpanjian College of Health Sciences, American University of Armenia, Yerevan, Armenia; 3 Department of Hygiene and Ecology, Yerevan State Medical University, Yerevan, Armenia; 4 Department of Emergency Medicine, Yale School of Medicine, New Haven, USA; 5 Department of Pediatrics, Inova L.J. Murphy Children’s Hospital, Falls Church, USA

**Keywords:** Prehospital care, Emergency systems, Electronic health records, Armenia

## Abstract

**Background:**

Emergency medical services (EMS) are paramount to boosting health indices in lower-middle income countries (LMICs); however, lack of uniform data collection and analysis hinders system improvement efforts. In the present study, we describe patterns of EMS utilization in the Republic of Armenia and provide key insight into the quality of digital data collection methods.

**Results:**

For calls logged in the capital city, Yerevan, the majority had at least one missing field. The predominant complaint was high blood pressure among adults (34.4%) and fever among pediatrics (65.9%). A majority of patients were female (57.6%), adults (90.2%), and not transported to a hospital (85.0%). In the rural provinces, the data was largely intact. The predominant complaints were unspecified acute condition (27.4%) and high blood pressure (26.2%) among adults, and fever (43.9%) and unspecified acute condition (22.1%) among pediatrics. A majority of patients were female (57.1%), adults (94.2%), and not transported to a hospital (78.9%).

**Conclusions:**

Our study reveals that the majority of calls to the EMS system are for concerns not needing in-hospital treatment and for acute exacerbation of chronic conditions. Our study also provides a critical foundation for the improvement of EMS systems in Armenia and in other nations in transition. The Locator software has the potential to be a valuable tool to the MoH if it is improved for surveillance purposes, and future synchronization of digital systems would provide easy access to critical information on population health needs and the effectiveness of public health interventions.

**Supplementary Information:**

The online version contains supplementary material available at 10.1186/s12245-024-00644-y.

## Background

Emergency Medical Services (EMS) are often the first line of contact of acutely ill and injured patients. A well-organized emergency medical system both directly maximizes optimal outcomes for patients and leads to early recognition and diagnosis of acute life-threatening conditions, paired with timely access to life saving medical care [1, 2]. EMS in lower-middle income countries (LMICs), including ambulance services, remain underdeveloped, underfunded, and heterogenous [3, 4]. As a result, EMS has been identified as an area of healthcare in LMICs that requires significant improvement to boost overall health indices [2, 3]. Strengthening prehospital care alone has the potential of reducing trauma mortality by up to 50% in some countries [5]. However, a lack of uniform data collection hinders efforts to reform national EMS policies [2, 3, 6–8]. The challenges presented to LMICs in data collection and analysis of EMS are unique and significant [7].

The Republic of Armenia is a landlocked nation located in the South Caucasus region of Asia, sharing borders with Turkey, Georgia, Iran, and Azerbaijan. Armenia has been a state in transition since its independence from the USSR in 1991, and continues to struggle with modernization of healthcare systems, including EMS, due to the deeply rooted legacies of outdated Soviet systems [2]. Armenia was considered an LMIC until 2018 and is now categorized as an upper-middle income country (UMIC) by the World Bank [9]. This improved economic position has allowed Armenia to concentrate on programmatic innovation and amelioration of healthcare.

As part of these efforts, the Ministry of Health of Armenia (MoH) has taken steps toward digitization of EMS data. To our knowledge, there are no published studies that describe EMS utilization or the quality of EMS digital data collection in Armenia. Our study begins to address these gaps in the literature. We conducted a retrospective review of data entered through the Locator™ software from January 2016 to July 2022. We describe patterns of EMS utilization in Armenia, focusing on patient demographics, chief complaints, and hospitalizations, as well as ambulance response timing.

## Methods

### Study setting

Armenia has a population of just under 3 million people, of which 39% reside in the capital city of Yerevan and the remaining 61% reside in Armenia’s rural provinces, known as *marzes* [10, 11]. Gyumri and Vanadzor, the second and third most populous cities, are home to 5.3% and 3.6% of the population, respectively.

**Fig. 1:**
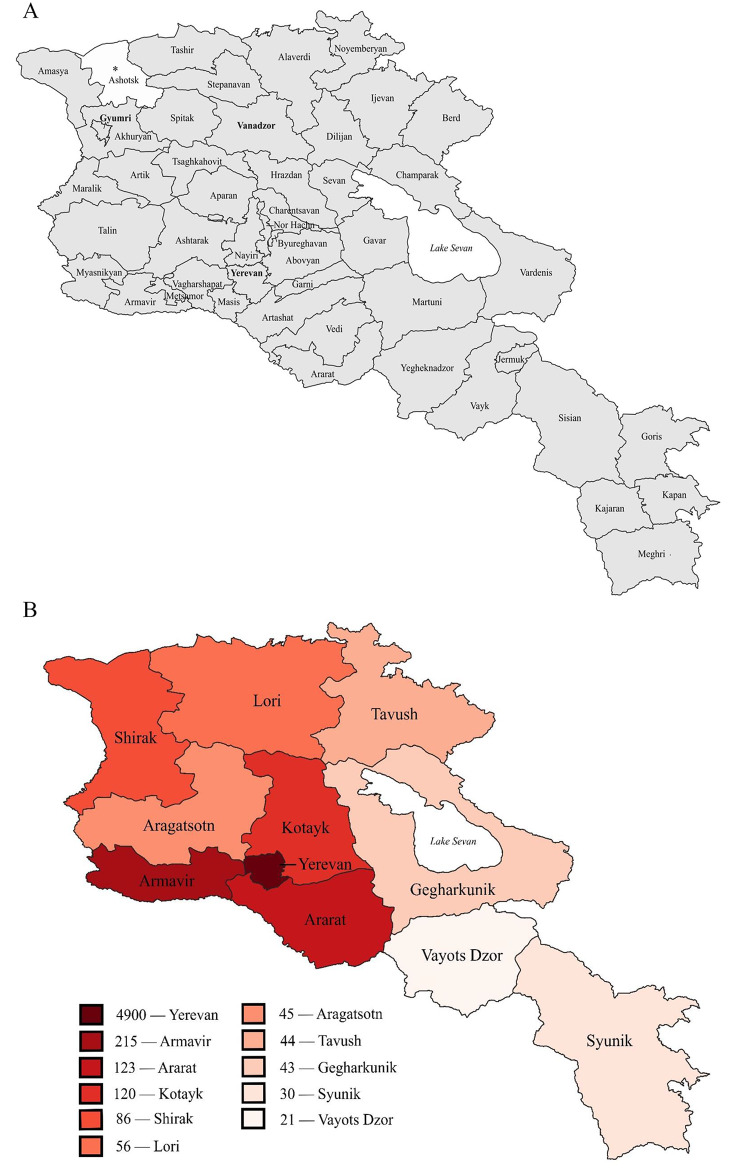
Geographic Coverage by Ambulance Station and Population Density by Marz. *Note.* (**a**) Estimation of geographic coverage by state-funded ambulance stations [27], utilizing a digital file provided by A. Nazaryan in August 2023 (**b**) Population density (person per square kilometer) across Armenia’s 10 marzes and capital city Yerevan [28, 29]. * Private ambulance coverage only

Armenia’s EMS system follows the Franco-German (stay and stabilize) model, in which an ambulance team, or *brigade*, typically consisting of a physician, a nurse, and a driver, is dispatched to provide care on-scene or “out-of-hospital” [1, 2, 6]. Ambulance services are subsidized by the Armenian government, making them completely free of charge; however, there are some private hospitals in Yerevan which operate their own ambulances and do require payment for transport [2]. As of October 2022, a total of 51 active brigades operate through the seven ambulance stations in Yerevan, 14 through the central station in Gyumri, and 5 through the central station in Vanadzor, according to an email from N. Pahlevanyan (npahlevanyan@moh.am) in February 2023. The remaining 109 brigades cover the 44 state-funded ambulance stations in rural Armenia, making geographic coverage challenging in many areas (See Fig. 1a). During the SARS-CoV-2 (COVID-19) pandemic, an eighth temporary substation in Yerevan was opened which responded only to callers who had already been diagnosed with COVID-19 [12].

### Study design

This is a descriptive retrospective cohort study utilizing a de-identified version of the Locator database, provided by the MoH, covering the cities of Yerevan, Gyumri, and Vanadzor, as well as Armenia’s 10 rural marzes. The study spans from January 1, 2016 to July 31, 2022.

### Locator software

In 2011, a software developed by Locator™ was implemented in every state-funded ambulance station in Armenia, allowing EMS dispatchers to submit call information online (Locator, n.d.) in a format designed to mirror paper call sheets. The software is updated in real-time and organizes input variables from calls logged across the country into a single master database, which is accessible to the MoH. It additionally features GPS-location capabilities, allowing the MoH to keep track of active ambulances at any time.

The Locator database is utilized by all 54 state-funded ambulance stations (including the temporary eighth station opened in Yerevan during the COVID-19 pandemic), as well as 22 primary care facilities which input data from home visits. Due to the aims of the present study, we excluded data input by these non-emergency units from analysis.

By design, the software requires users to select at least one of three broad categories (acute condition, chronic illness exacerbation, and accident) prior to selecting a specific chief complaint for each call. Diagnoses are input as free text. Hospitalization data indicates whether a patient is transported to a hospital (yes or no) but does not specify which hospital they are taken to.

### Data analysis

Descriptive statistics were analyzed using IBM SPSS Statistics, version 28.0.1.1 (14). Figures were generated using Microsoft Excel, version 16.68 and QGIS software, version 3.26.3. Non-numerical values in the database were provided in the Armenian language, which the authors of this paper read and translated.

Chief complaints listing both broad and specific categories (e.g., “chronic illness exacerbation, diabetes”) were simplified to report only the specific complaints (e.g., “diabetes”). Nonsensical outliers in age and date columns were removed and counted as missing information. Age was limited to the range of 0-100 years old. Time to arrive, time spent on scene, and call duration variables were limited to 24 h, which eliminated errors made in recording the date of a call. Time to arrive was defined as the time (measured in minutes) between ambulance departure and arrival to the scene. Time spent on scene was defined as the time between arrival to the scene and call completion. Call duration was defined as the time between ambulance departure and call completion. Seasons were classified as Winter (December-February), Spring (March-May), Summer (June-August), and Fall (September-November).

Of the 54 stations included in the database, 14 were found to have significant gaps in data, with 0 calls reported for at least 12 consecutive months and up to over 3 consecutive years. The data deficiencies were verified with the MoH and the administration of the Gyumri ambulance station. These 14 stations, which are situated near the cities of Aparan, Masis, Vedi, Vardenis, Stepanavan, Tashir, Spitak, Alaverdi, Meghri, Gyumri, Akhuryan, Artik, Vayk, and Dilijan, were subsequently removed from the database (Fig. 1a).

## Results

A total of 2,996,426 calls were analyzed. The majority of calls (*n* = 1,864,289; 62.2%) were logged in Yerevan (Table 1). The majority of data had at least one missing field (83.6% gender, 40.0% age, 50.1% chief complaints, 83.5% diagnoses, 20.8% hospitalization, 33.0% ambulance departure time, 33.0% arrival to scene time, and 3.2% call completion time). The remaining calls (*n* = 1,132,137; 37.8%) occurred in the marzes (3.9% missing gender, 11.4% age, 4.2% chief complaints, 20.3% diagnoses, 1.8% hospitalization, 1.0% ambulance departure time, 1.0% arrival to scene time, and 0.7% call completion time).

**Table 1:** Demographics

	*n* (%)
Gender*	
Male	596,488 (42.8)
Female	796,836 (57.2)
Age**	
Years (mean, sd)	53.1 (22.3)
< 1 year	48 (0.0)
1–4 years	65,504 (3.0)
5–11 years	64,654 (2.9)
12–17 years	45,059 (2.1)
18–39 years	427,472 (19.5)
40–59 years	598,991 (27.3)
60 + years	995,755 (45.3)
**Marz**	
Aragatsotn	79,179 (2.6)
Ararat	112,452 (3.8)
Armavir	215,477 (7.2)
Gegharkunik	111,918 (3.7)
Kotayk	243,236 (8.1)
Lori	147,811 (4.9)
Shirak	21,920 (0.7)
Syunik	95,753 (3.2)
Tavush	63,332 (2.1)
Vayots Dzor	41,059 (1.4)
Yerevan	1,864,289 (62.2)
**Year**	
2016	400,669 (13.4)
2017	384,121 (12.8)
2018	406,639 (13.6)
2019	437,678 (14.6)
2020	537,078 (17.9)
2021	537,999 (18.0)
2022	292,242 (9.8)
**Season**	
Winter	791,407 (26.4)
Spring	759,211 (25.3)
Summer	723,800 (24.2)
Fall	722,008 (24.1)
**Time of Call**	
Midnight − 0759	584,204 (19.5)
0800–1559	1,025,335 (34.2)
1600–2359	1,386,887 (46.3)
**Chief Complaints** ^**1**^	
***Trauma***	
Injuries (unspecified)	49,914 (2.5)
Bleeding	21,171 (1.1)
Road traffic injury	16,749 (0.8)
Accident (unspecified)	7308 (0.4)
Road traffic injury (pedestrian)	3193 (0.2)
Fall	3077 (0.2)
Burn	2308 (0.1)
Dog bite	1286 (0.1)
Firearm injury	654 (< 0.1)
Vein incision	493 (< 0.1)
Electrocution	394 (< 0.1)
Stabbing	353 (< 0.1)
Hanging (suicide)	257 (< 0.1)
Drowning	133 (< 0.1)
***Non-Trauma***	
High Blood Pressure	532,738 (26.4)
Acute condition (unspecified)	363,855 (18.1)
Fever	260,743 (12.9)
Abdominal pain	142,749 (7.1)
Chest pain	128,007 (6.4)
Shortness of breath	116,405 (5.8)
Loss of consciousness	72,867 (3.6)
Cardiovascular disease	44,338 (2.2)
Chronic disease exacerbation (unspecified)	43,918 (2.2)
Cancer	24,533 (1.2)
Cerebrovascular disease	22,793 (1.1)
Psychiatric issue	21,679 (1.1)
Respiratory disorder	21,344 (1.1)
Seizures	16,007 (0.8)
Diabetes	13,656 (0.7)
Pregnancy issue	6647 (0.3)
Alcohol poisoning	5580 (0.3)
Childbirth/labor	4105 (0.2)
Food poisoning	2987 (0.1)
Medicine poisoning	2238 (0.1)
Snake and bug bites	1749 (0.1)
Chemical poisoning	1499 (0.1)
Carbon monoxide poisoning	1234 (0.1)
***Transport***	
Transport	73,618 (3.7)
Transport of deceased	63,715 (3.2)
Hospitalized***	
No	2,132,137 (82.4)
Yes	456,795 (17.6)
Time to Reach Destination****	
Minutes (mean, sd)	8.1 (14.8)
≤ 5 min	1,175,162 (49.6)
6 to 10 min	835,839 (35.3)
11 to 20 min	266,623 (11.3)
21 to 30 min	47,884 (2.0)
30 + minutes	43,436 (1.8)
Time Spent at Destination*****	
Minutes (mean, sd)	33.4 (45.8)
≤ 10 min	253,317 (10.8)
11 to 20 min	615,608 (26.2)
21 to 30 min	660,828 (28.1)
31 to 40 min	337,281 (14.4)
41 to 50 min	169,856 (7.2)
51 to 60 min	97,800 (4.2)
61 to 120 min	166,584 (7.1)
121 to 180 min	26,285 (1.1)
181 + minutes	20,468 (0.9)
Total Call Duration******	
Minutes (mean, sd)	41.5 (51.8)
≤ 10 min	147,767 (6.3)
11 to 20 min	321,472 (13.7)
21 to 30 min	641,418 (27.3)
31 to 40 min	480,805 (20.5)
41 to 50 min	278,531 (11.9)
51 to 60 min	160,739 (6.8)
61 to 120 min	250,652 (10.7)
121 to 180 min	37,425 (1.6)
181 + minutes	29,246 (1.2)
**TOTAL**	2,996,426 (100)

^1^Could choose more than 1 option

*n = 1,393,324

**n = 2,197,483

***n = 2,588,932

****n = 2,368,944

*****n = 2,348,027

******n = 2,348,055

In Yerevan, 57.6% of patients were female, 90.2% were adults, and 85.0% were not transported to a hospital. In the marzes, 57.1% of patients were female, 94.2% were adults, and 78.9% were not transported to a hospital. In Yerevan, 6.9% of complaints were classified as broad (See Methods), and lacked specificity (Appendix A), compared to 32.4% in the marzes. Overall, call volume was highest in the later hours of the day, 1600–2359, (46.3%) and lowest in the early morning, midnight − 0759, (19.5%) (Table 1).

Top five chief complaints varied by age, gender, and location (Tables 2a and 2b). In Yerevan, the predominant complaint among all adults included high blood pressure (34.4%), and the most common complaint among pediatrics was fever (65.9%).

**Table 2a:** Top 5 Complaints by Age in the Marzes

		Chief Complaint Ranking*					
		Male		Female		Total**	
		Complaint	*n* (%)	Complaint	*n* (%)	Complaint	*n* (%)
**Age Group**	**< 1**	Acute condition (unspecified)	11 (50.0)	Acute condition (unspecified)	8 (47.1)	Acute condition (unspecified)	20 (50.0)
		Fever	7 (31.8)	Fever	7 (41.2)	Fever	14 (35.0)
		Seizures	2 (9.1)	Road traffic injury	1 (5.9)	Road traffic injury	2 (5.0)
		Road traffic injury	1 (4.5)	Transport	1 (5.9)	Seizures	2 (5.0)
		Injuries (unspecified)	1 (4.5)	-	-	Injuries (unspecified)	1 (2.5)
		Chest pain	1 (4.5)	-	-	Transport	1 (2.5)
		-	-	-	-	Chest pain	1 (2.5)
	**1 to 4**	Fever	5675 (55.1)	Fever	4067 (56.3)	Fever	10,084 (55.5)
		Acute condition (unspecified)	1885 (18.3)	Acute condition (unspecified)	1292 (17.9)	Acute condition (unspecified)	3323 (18.3)
		Transport	695 (6.7)	Transport	430 (5.9)	Transport	1205 (6.6)
		Shortness of breath	460 (4.5)	Abdominal pain	368 (5.1)	Abdominal pain	823 (4.5)
		Abdominal pain	443 (4.3)	Shortness of breath	271 (3.7)	Shortness of breath	762 (4.2)
	**5 to 11**	Fever	5641 (48.1)	Fever	4079 (49.5)	Fever	10,032 (48.6)
		Acute condition (unspecified)	2239 (19.1)	Acute condition (unspecified)	1561 (18.9)	Acute condition (unspecified)	3974 (19.2)
		Abdominal pain	1060 (9.0)	Abdominal pain	869 (10.5)	Abdominal pain	1976 (9.6)
		Injuries (unspecified)	618 (5.3)	Transport	357 (4.3)	Transport	951 (4.6)
		Transport	546 (4.7)	Injuries (unspecified)	299 (3.6)	Injuries (unspecified)	941 (4.6)
	**12 to 17**	Fever	2935 (31.1)	Acute condition (unspecified)	2478 (31.7)	Acute condition (unspecified)	5187 (29.1)
		Acute condition (unspecified)	2545 (27.0)	Fever	1682 (21.5)	Fever	4779 (26.8)
		Abdominal pain	862 (9.1)	Abdominal pain	1385 (17.7)	Abdominal pain	2323 (13.0)
		Injuries (unspecified)	635 (6.7)	Loss of consciousness	468 (6.0)	Loss of consciousness	891 (5.0)
		Loss of consciousness	382 (4.0)	Shortness of breath	303 (3.9)	Injuries (unspecified)	873 (4.9)
	**18 to 39**	Acute condition (unspecified)	27,778 (36.6)	Acute condition (unspecified)	38,104 (41.2)	Acute condition (unspecified)	67,647 (39.2)
		Fever	10,013 (13.2)	Fever	9400 (10.2)	Fever	20,047 (11.6)
		Abdominal pain	6353 (8.4)	Abdominal pain	9137 (9.9)	Abdominal pain	15,826 (9.2)
		High blood pressure	5809 (7.6)	High blood pressure	7516 (8.1)	High blood pressure	13,733 (7.9)
		Chest pain	4709 (6.2)	Chest pain	5131 (5.5)	Chest pain	10,028 (5.8)
	**40 to 59**	Acute condition (unspecified)	34,329 (29.2)	High blood pressure	50,806 (32.2)	Acute condition (unspecified)	81,605 (29.0)
		High blood pressure	23,786 (20.2)	Acute condition (unspecified)	45,084 (28.6)	High blood pressure	76,047 (27.0)
		Chest pain	11,511 (9.8)	Abdominal pain	9694 (6.1)	Chest pain	20,343 (7.2)
		Shortness of breath	7206 (6.1)	Chest pain	8433 (5.3)	Abdominal pain	17,131 (6.1)
		Abdominal pain	7101 (6.0)	Chronic disease exacerbation (unspecified)	8363 (5.3)	Fever	15,091 (5.4)
	**60 +**	High blood pressure	41,673 (23.7)	High blood pressure	103,544 (38.6)	High blood pressure	148,313 (32.6)
		Acute condition (unspecified)	41,243 (23.5)	Acute condition (unspecified)	55,414 (20.6)	Acute condition (unspecified)	100,033 (22.0)
		Shortness of breath	15,735 (9.0)	Shortness of breath	15,623 (5.8)	Shortness of breath	32,103 (7.0)
		Chest pain	12,655 (7.2)	Cardiovascular disease	14,712 (5.5)	Chest pain	26,159 (5.7)
		Cardiovascular disease	10,441 (5.9)	Chest pain	12,917 (4.8)	Cardiovascular disease	25,454 (5.6)

*Percentages and totals are based on calls reporting at least 1 chief complaint

**Includes calls that did not report gender

**Table 2b:** Top 5 Complaints by Age in Yerevan

		Chief Complaint Ranking*					
		Male		Female		Total**	
		Complaint	*n* (%)	Complaint	*n* (%)	Complaint	*n* (%)
**Age Group**	**< 1**	None Listed	-	-	-	-	-
	**1 to 4**	Fever	4332 (72.7)	Fever	3479 (75.6)	Fever	21,891 (73.9)
		Transport	346 (5.8)	Transport	299 (6.5)	Transport	2148 (7.2)
		Shortness of breath	280 (4.7)	Abdominal pain	201 (4.4)	Abdominal pain	1291 (4.3)
		Abdominal pain	276 (4.6)	Injuries (unspecified)	164 (3.6)	Injuries (unspecified)	1042 (3.5)
		Injuries (unspecified)	205 (3.4)	Seizures	131 (2.8)	Acute condition (unspecified)	836 (2.8)
	**5 to 11**	Fever	3312 (65.3)	Fever	2487 (67.1)	Fever	17,770 (66.6)
		Abdominal pain	613 (12.1)	Abdominal pain	479 (12.9)	Abdominal pain	3619 (13.6)
		Injuries (unspecified)	318 (6.3)	Transport	187 (5.0)	Transport	1445 (5.4)
		Transport	260 (5.1)	Injuries (unspecified)	153 (4.1)	Injuries (unspecified)	1261 (4.7)
		Shortness of breath	151 (3.0)	Acute condition (unspecified)	98 (2.6)	Acute condition (unspecified)	758 (2.8)
	**12 to 17**	Fever	1028 (47.6)	Fever	579 (35.8)	Fever	6218 (46.8)
		Abdominal pain	293 (13.6)	Abdominal pain	436 (27.0)	Abdominal pain	2744 (20.6)
		Injuries (unspecified)	246 (11.4)	Loss of consciousness	172 (10.6)	Injuries (unspecified)	906 (6.8)
		Loss of consciousness	109 (5.0)	Injuries (unspecified)	89 (5.5)	Loss of consciousness	690 (5.2)
		Transport	99 (4.6)	Acute condition (unspecified)	69 (4.3)	Transport	631 (4.7)
	**18 to 39**	Fever	3505 (25.5)	Fever	3522 (22.3)	Fever	29,205 (29.1)
		High Blood Pressure	1925 (14.0)	Abdominal pain	2791 (17.7)	Abdominal pain	16,238 (16.2)
		Abdominal pain	1611 (11.7)	High Blood Pressure	2331 (14.8)	High Blood Pressure	14,435 (14.4)
		Chest pain	1396 (10.2)	Loss of consciousness	1298 (8.2)	Chest pain	7629 (7.6)
		Acute condition (unspecified)	847 (6.2)	Acute condition (unspecified)	1155 (7.3)	Acute condition (unspecified)	6818 (6.8)
	**40 to 59**	High Blood Pressure	5354 (29.6)	High Blood Pressure	12,245 (51.4)	High Blood Pressure	55,972 (39.7)
		Chest pain	2551 (14.1)	Fever	1884 (7.9)	Fever	17,656 (12.5)
		Shortness of breath	2100 (11.6)	Chest pain	1824 (7.7)	Chest pain	15,383 (10.9)
		Fever	1419 (7.8)	Abdominal pain	1738 (7.3)	Abdominal pain	11,797 (8.4)
		Abdominal pain	1386 (7.6)	Shortness of breath	1412 (5.9)	Acute condition (unspecified)	8221 (5.8)
	**60 +**	High Blood Pressure	6653 (29.1)	High Blood Pressure	22,228 (51.2)	High Blood Pressure	102,702 (39.2)
		Shortness of breath	4143 (18.1)	Shortness of breath	4238 (9.7)	Transport of deceased	26,913 (10.3)
		Chest pain	2366 (10.4)	Chest pain	3134 (7.2)	Fever	24,238 (9.2)
		Acute condition (unspecified)	1631 (7.1)	Acute condition (unspecified)	2691 (6.2)	Chest pain	19,858 (7.6)
		Loss of consciousness	1576 (6.9)	Abdominal pain	2278 (5.2)	Shortness of breath	19,311 (7.4)

*Percentages and totals are based on calls reporting at least 1 chief complaint

**Includes calls that did not report gender

In the marzes, the largest patient demographic was women over 60 (28.3%) followed by men over 60 (18.6%). The most common complaints among all adults were listed as unspecified acute condition (27.4%) and high blood pressure (26.2%). High blood pressure made up 7.9% of complaints in young adults (18–39), 27.0% in middle aged adults (40–59), and 32.6% in older adults (60+) (Table 2a.). For women over 60, high blood pressure was listed as a complaint in 38.6% of calls. Among pediatrics, the most common complaints were fever (43.9%) and unspecified acute condition (22.1%). Overall, excluding blood pressure, another 13.8% of calls in the marzes included complaints of chronic illness exacerbations, 4.5% involved trauma, 2.5% were transports of patients to healthcare facilities, and 0.8% were transports of the deceased to morgues (Appendix A).

Ambulances arrived on scene within 10 min of departure for the majority of calls in both Yerevan and the marzes (90.4% and 78.8%, respectively) and arrived within 30 min for 99.4% and 96.8% (Appendix B). Responders spent fewer than 31 min on scene for the majority of calls in both Yerevan and the marzes (56.8% and 74.3%) and fewer than 41 min for 72.6% and 87.1%.

**Fig. 2:**
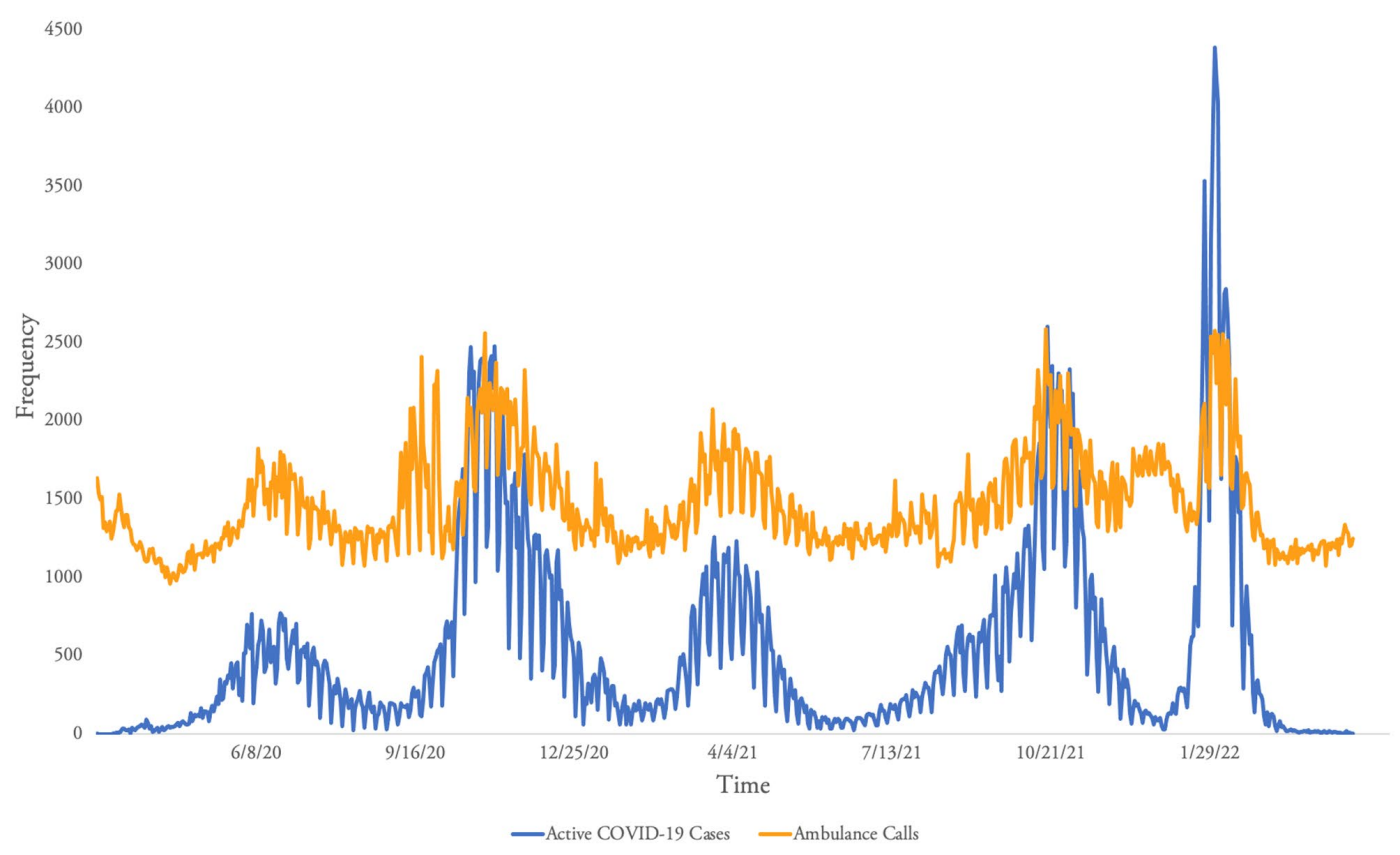
Daily Ambulance Calls and COVID-19 Cases. *Note.* Trends of daily ambulance calls and active COVID-19 cases from March, 2020 to April, 2022 [30]

Fluctuations in the total daily national ambulance call trends matched the pattern of daily new positive COVID-19 cases from March, 2020 to April, 2022 (Fig. 2). Yerevan’s eighth temporary substation, which responded only to COVID-19 patients, ran from December 2020 until May 2021 and logged 484 calls.

### Additional findings of missing data

Analysis revealed that data from most stations in both the marzes and Yerevan had some deficiencies. Notably, the stations in Amasya, Tsaghkahovit, and Jermuk had 465, 377, and 104 non-consecutive days with 0 calls logged, respectively. The rest of the stations had fewer than 85 days with 0 calls logged. The database as a whole lacks data from August 31, 2017 and August 21, 2018.

## Discussion

Our study is the first to describe patterns of ambulance usage in Armenia, and the first to analyze and assess the implementation of the electronic EMS Locator software system.

### Epidemiology of calls

In both Yerevan and the marzes, we found that the predominant complaint among adults, outside of the broad “acute condition” category, was high blood pressure. These findings are consistent with prior reports describing the considerable population of adults with uncontrolled hypertension in Armenia [13]. Remarkably, to our knowledge, no other study has listed hypertension or high blood pressure as a leading chief complaint among patients utilizing ambulance services. In fact, several studies conducted in low-income and LMIC countries report maternal care and pregnancy complications, trauma, and interfacility transports as leading complaints [14–18]. In the present study, these complaints were listed in a small percentage of calls (Table 1). It is possible that those patients drive to healthcare facilities in private vehicles and don’t contact EMS in Armenia.

In both Yerevan and the marzes, we found that the predominant complaint among pediatric patients was fever. Though international EMS literature largely lacks pediatric data, this finding has been documented in India [19]; however, that study also documented high percentages of trauma and respiratory complaints among pediatrics, which were not found in the present study. Another study, conducted in Belgium, finds a low percentage of fever as a chief complaint among pediatrics, and instead identifies neurologic issues (specifically seizures) and trauma as the top complaints [20].

Notably, overall rates of hospitalization for ambulance calls in Armenia are much lower than those reported in other countries [15, 21, 22]. Additionally, prehospital care in Armenia services an older population than was found in Saudi Arabia, Zimbabwe, and Ethiopia [14, 16, 21]; however, our age findings were similar to studies conducted in Japan and Iran [15, 22]. These results are consistent with Armenia’s age distribution, as 13% of the population are adults over the age of 65, compared to 3% in Saudi Arabia, Zimbabwe, and Ethiopia [23].

As expected, COVID-19 cases are associated with increases in frequencies of national daily ambulance calls (Fig. 2). This illustrates how shifting population needs can cause immediate changes in utilization patterns, as reflected in the database.

The response times found in our study are longer than the international standards reported in the literature (Appendix A) [2]. This could be explained by the fact that many stations in Armenia’s marzes have very few active ambulances and need to cover large geographic areas (Fig. 1).

The high frequency of calls related to blood pressure and chronic illnesses constitute an extraordinarily heavy burden on the EMS system. Wide-spread implementation of hypertension screenings, expanded government coverage of hypertension medications, and increased access to primary care follow-ups for patients with chronic illnesses will likely reduce the number of emergencies associated with these conditions. Reducing these burdens will increase ambulance availability for acute emergencies that require timely intervention and transport, particularly in large geographic areas covered by few ambulances (Fig. 1). Moreover, in addition to responding to emergency calls, ambulances and their staff are utilized to transport patients to healthcare facilities and the deceased to morgues. While the MoH may consider increasing the total number of active brigades to accommodate these burdens on the EMS system, it may prove more beneficial to reallocate these non-emergency responsibilities. Additionally, our analysis shows that call frequencies vary by time of day. Using this information, the number of active brigades at a given ambulance station could be scheduled so that they increase during peak volume hours and decrease during off-peak hours.

It is likely that the ambulance system is misused, given that it is a cost-free and convenient service. The MoH may consider the implementation of mobile urgent care clinics, after-hours assistance at primary care facilities, medical hotlines, and protocolized medical direction from dispatchers as measures to alleviate strains on current system capacity, reduce misuse, and improve patient outcomes.

### Limitations

Our study is limited by the missing information in the database analyzed. This might impact the generalizability of our data as well as the significance of conclusions. Large gaps in the data resulted in the exclusion of 14 ambulance stations in the present study. Analysis of the remaining stations subsequently revealed that many clinically relevant columns were left largely unfilled for calls logged in Yerevan. Moreover, even within those cells that did contain information, we found that the quality of data was often poor due to either a lack of specificity or a lack of standardization of answers. For example, many calls listed broad chief complaints without including a specific complaint. The lack of clarification in these cases suggests that the appropriate specific categories are not available as options on the Locator site or the paper call sheets. Additionally, hospitalization was reported as a binary (yes or no) without including information about where patients were transported. Finally, the free texted diagnosis column introduces several unnecessary challenges due to a lack of standardization of terminology, accidental typos, and mixed usage of Armenian, Latin, and Russian letters. The issues outlined in the present study are likely to be encountered anywhere a new digital data collection system is implemented, making the study findings particularly relevant for other nations in transition.

### Opportunities for improvement

The Locator software has the potential to be a valuable tool for the MoH if it is improved for surveillance purposes. Gaps in information within clinically relevant columns could be solved by making them required entries. Errors in manual inputs of numerical information could be avoided by imposing entry restrictions (such as minimums and maximums for the age variable) or by prompting the user with a pop-up notification when an inputted variable falls outside of the normal bounds. Locator’s free texted diagnosis entry should require users to select from a list of associated International Classification of Diseases (ICD) codes. Additionally, given the high frequency of complaints listed as broad categories, the options on both the MoH paper call sheets and the Locator software should be expanded to include other common specific complaints.

The MoH should consider implementing periodic inspections of the database to ensure calls are being logged into Locator, which would prevent the large consecutive gaps in data found in our study. Future research should include reliability checks comparing the accuracy of Locator data with information recorded on paper call sheets to best describe the current state of EMS record digitization in Armenia.

Future efforts to digitize medical records in Armenia should proceed with the intention of creating multi-purpose databases. Ideal data collection software must be adaptable for public health and clinical surveillance purposes. A model prehospital digital database [7] would allow researchers to access and extract data relevant to understanding community-level health needs and the quality and accessibility of care, among other factors. Such information would facilitate the prioritization and implementation of interventions aimed at improving population health conditions, including myocardial infarction, stroke, and trauma. Moreover, merging existing digital systems in Armenia would synchronize prehospital, hospital, and out of hospital care information, allowing for easy bidirectional access to records by both patients and their physicians in addition to providing a de-identified master database for surveillance purposes.

Medical record digitization has the potential to improve efficiency, organization, quality of care, and data accessibility, quality, and accuracy, among other important factors, in healthcare settings across the world [24]. However, there are barriers to successfully implementing digital systems, and these may instead lead to negative outcomes. Insufficient training, lack of baseline technological literacy, and lack of motivation to properly use digital systems by ambulance dispatchers and medical staff will pose a large challenge for the MoH. To ensure that digital systems are implemented in a way that optimizes their positive effects, the MoH must incorporate standardized and periodic staff training to address relevant gaps in medical knowledge, technological skills, and comprehension of the benefits of record digitization.

Finally, the MoH must take steps to improve its EMS capacity overall. The recent influx of refugees from the neighboring Nagorno-Karabakh (or Artsakh) region introduces new challenges for Armenia’s healthcare system, particularly because this refugee population has faced a blockade leading to a dearth of essential medications, starvation and malnutrition, as well as mental and physical trauma [25, 26]. These health issues will need to be addressed and may pose long-term strains on Armenia’s healthcare capacity.

## Conclusions

This study provides a critical foundation for the improvement of EMS systems in Armenia and in other nations in transition. The electronic EMS Locator database provides opportunities for intervention at the community level to improve the burden of noncommunicable diseases, such as hypertension. Our study also finds many opportunities to improve the utilization of the electronic database for continued quality assurance and safety initiatives. Further studies linking Locator to in-hospital database systems can be used to assess outcomes of patient transfers to hospitals via ambulance and will provide important information about the impact of pre-hospital care provided.

### Electronic supplementary material

Below is the link to the electronic supplementary material.

Supplementary Material 1

Supplementary Material 2

## Data Availability

Locator™ ambulance call data and information regarding geographic coverage of ambulance stations were acquired through the MoH and are not publicly available files. The study utilizes data published by The World Bank, available from https://data.worldbank.org/indicator/SP.POP.TOTL? locations=AM and https://data.worldbank.org/indicator/SP.POP.65UP.TO; [10, 23] World Population Review, available from https://worldpopulationreview.com/countries/cities/armenia; [21] ArcGIS Online, available from https://data-download-gfw.hub.arcgis.com/;[27] Humanitarian Data Exchange, available from https://data.humdata.org/dataset/cod-ab-arm; [28] the Statistical Committee of the Republic of Armenia, available from https://armstat.am/file/doc/99534458.pdf; [29] and the National Center for Disease Control Armenia, available from https://ncdc.am/coronavirus/confirmed-cases-by-days/.[30].

## Declarations

### Disclaimers

The paper is the product of professional research and represents the views of the authors. The opinions presented are not official positions of affiliated institutions or funders.

## Abbreviations

EMS: Emergency Medical Services
LMIC: Lower–middle income countries
UMIC: Upper–middle income country
MoH: Ministry of Health of Armenia
