# Supplementary material for: Emergency medical services in Armenia: national call trends and future directions
Source: Int J Emerg Med. 2024 May 16;17:65. doi: 10.1186/s12245-024-00644-y (PMC11097514; doi:10.1186/s12245-024-00644-y)
Supplement: Supplementary file 1 — Supplementary Material 1 [file 12245_2024_644_MOESM1_ESM.docx]

**Appendix A**

*Chief Complaints by Location, All Ages*

|  | **Location** | | **Total n(%)** |
| --- | --- | --- | --- |
| **Chief Complaint^1^** | **All Marzes n(%)** | **Yerevan n(%)** |  |
| ***Trauma*** |  |  | |
| Injuries (unspecified) | 20986 (1.9) | 28928 (3.1) | 49914 (2.5) |
| Bleeding | 9797 (0.9) | 11374 (1.2) | 21171 (1.1) |
| Road traffic injury | 9285 (0.9) | 7464 (0.8) | 16749 (0.8) |
| Accident (unspecified) | 3478 (0.3) | 3830 (0.4) | 7308 (0.4) |
| Road traffic injury (pedestrian) | 873 (0.1) | 2320 (0.2) | 3193 (0.2) |
| Fall | 2263 (0.2) | 814 (0.1) | 3077 (0.2) |
| Burn | 708 (0.1) | 1600 (0.2) | 2308 (0.1) |
| Dog bite | 424 (<0.1) | 862 (0.1) | 1286 (0.1) |
| Firearm injury | 612 (0.1) | 42 (<0.1) | 654 (<0.1) |
| Vein incision | 154 (<0.1) | 339 (<0.1) | 493 (<0.1) |
| Electrocution | 180 (<0.1) | 214 (<0.1) | 394 (<0.1) |
| Stabbing | 144 (<0.1) | 209 (<0.1) | 353 (<0.1) |
| Hanging (suicide) | 194 (<0.1) | 63 (<0.1) | 257 (<0.1) |
| Drowning | 115 (<0.1) | 18 (<0.1) | 133 (<0.1) |
| ***Non-Trauma*** |  | |  |
| High Blood Pressure | 263919 (24.3) | 268819 (28.9) | 532738 (26.4) |
| Acute condition (unspecified) | 304988 (28.1) | 58867 (6.3) | 363855 (18.1) |
| Fever | 93881 (8.7) | 166862 (18.0) | 260743 (12.9) |
| Abdominal pain | 64417 (5.9) | 78332 (8.4) | 142749 (7.1) |
| Chest pain | 61941 (5.7) | 66066 (7.1) | 128007 (6.4) |
| Shortness of breath | 62272 (5.7) | 54133 (5.8) | 116405 (5.8) |
| Loss of consciousness | 24982 (2.3) | 47885 (5.2) | 72867 (3.6) |
| Cardiovascular disease | 39240 (3.6) | 5098 (0.5) | 44338 (2.2) |
| Chronic disease exacerbation (unspecified) | 42777 (3.9) | 1141 (0.1) | 43918 (2.2) |
| Cancer | 13754 (1.3) | 10779 (1.2) | 24533 (1.2) |
| Cerebrovascular disease | 22116 (2.0) | 677 (0.1) | 22793 (1.1) |
| Psychiatric issue | 5376 (0.5) | 16303 (1.8) | 21679 (1.1) |
| Respiratory disorder | 19830 (1.8) | 1514 (0.2) | 21344 (1.1) |
| Seizures | 8954 (0.8) | 7053 (0.8) | 16007 (0.8) |
| Diabetes | 7007 (0.6) | 6649 (0.7) | 13656 (0.7) |
| Pregnancy issue | 2341 (0.2) | 4306 (0.5) | 6647 (0.3) |
| Alcohol poisoning | 1217 (0.1) | 4363 (0.5) | 5580 (0.3) |
| Childbirth/labor | 2191 (0.2) | 1914 (0.2) | 4105 (0.2) |
| Food poisoning | 470 (<0.1) | 2517 (0.3) | 2987 (0.1) |
| Medicine poisoning | 691 (0.1) | 1547 (0.2) | 2238 (0.1) |
| Snake and bug bites | 1236 (0.1) | 513 (0.1) | 1749 (0.1) |
| Chemical poisoning | 665 (0.1) | 834 (0.1) | 1499 (0.1) |
| Carbon monoxide poisoning | 943 (0.1) | 291 (<0.1) | 1234 (0.1) |
| ***Transport*** |  | |  |
| Transport | 27239 (2.5) | 46379 (5.0) | 73618 (3.7) |
| Transport of deceased | 8965 (0.8) | 54750 (5.9) | 63715 (3.2) |
| **Total*** | 1085082 | 929406 | 2014488 |
| ^1^Could choose more than 1 option  *Percentages and totals are based on calls reporting at least 1 chief complaint. | | | |
