# Supplementary material for: Emergency medical services in Armenia: national call trends and future directions
Source: Int J Emerg Med. 2024 May 16;17:65. doi: 10.1186/s12245-024-00644-y (PMC11097514; doi:10.1186/s12245-024-00644-y)
Supplement: Supplementary file 2 — Supplementary Material 2 [file 12245_2024_644_MOESM2_ESM.docx]

**Appendix B**

*Ambulance Timing by Location*

| **Time** | **Location** | | **Total* n(%)** |
| --- | --- | --- | --- |
|  | **All Marzes n(%)** | **Yerevan n(%)** |  |
| **Time to Reach Destination** | | | |
| *Minutes (mean, sd)* | 9.6 (18.4) | 6.7 (10.3) | 8.1 (14.8) |
| ≤5 minutes | 571826 (51.0) | 603336 (48.3) | 1175162 (49.6) |
| 6 to 10 minutes | 310639 (27.7) | 525200 (42.1) | 835839 (35.3) |
| 11 to 20 minutes | 169972 (15.2) | 96651 (7.7) | 266623 (11.3) |
| 21 to 30 minutes | 32377 (2.9) | 15507 (1.2) | 47884 (2.0) |
| 30+ minutes | 35342 (3.2) | 8094 (0.6) | 43436 (1.8) |
| Total | 1120156 | 1248788 | 2368944 |
| **Time Spent at Destination** | | | |
| *Minutes (mean, sd)* | 29.4 (33.0) | 37.0 (54.6) | 33.4 (45.8) |
| ≤10 minutes | 74282 (6.6) | 179035 (14.6) | 253317 (10.8) |
| 11 to 20 minutes | 385767 (34.5) | 229841 (18.7) | 615608 (26.2) |
| 21 to 30 minutes | 371420 (33.2) | 289408 (23.5) | 660828 (28.1) |
| 31 to 40 minutes | 142720 (12.8) | 194561 (15.8) | 337281 (14.4) |
| 41 to 50 minutes | 55950 (5.0) | 113906 (9.3) | 169856 (7.2) |
| 51 to 60 minutes | 27088 (2.4) | 70712 (5.8) | 97800 (4.2) |
| 61 to 120 minutes | 45201 (4.0) | 121383 (9.9) | 166584 (7.1) |
| 121 to 180 minutes | 9240 (0.8) | 17045 (1.4) | 26285 (1.1) |
| 181+ minutes | 6659 (0.6) | 13809 (1.1) | 20468 (0.9) |
| Total | 1118327 | 1229700 | 2348027 |
| **Total Call Duration** | | | |
| *Minutes (mean, sd)* | 39.1 (44.1) | 43.7 (57.8) | 41.5 (51.8) |
| ≤10 minutes | 17216 (1.5) | 130551 (10.6) | 147767 (6.3) |
| 11 to 20 minutes | 216599 (19.4) | 104873 (8.5) | 321472 (13.7) |
| 21 to 30 minutes | 375117 (33.5) | 266301 (21.7) | 641418 (27.3) |
| 31 to 40 minutes | 223808 (20.0) | 256997 (20.9) | 480805 (20.5) |
| 41 to 50 minutes | 114897 (10.3) | 163634 (13.3) | 278531 (11.9) |
| 51 to 60 minutes | 61533 (5.5) | 99206 (8.1) | 160739 (6.8) |
| 61 to 120 minutes | 80339 (7.2) | 170313 (13.8) | 250652 (10.7) |
| 121 to 180 minutes | 15327 (1.4) | 22098 (1.8) | 37425 (1.6) |
| 181+ minutes | 13506 (1.2) | 15740 (1.3) | 29246 (1.2) |
| Total | 1118342 | 1229713 | 2348055 |
| *Excludes those where time is missing/not reported. | | | |
